# Cross-Clade Protective Immune Responses to Influenza Viruses with H5N1 HA and NA Elicited by an Influenza Virus-Like Particle

**DOI:** 10.1371/journal.pone.0001501

**Published:** 2008-01-30

**Authors:** Rick A. Bright, Donald M. Carter, Corey J. Crevar, Franklin R. Toapanta, Jonathan D. Steckbeck, Kelly S. Cole, Niranjan M. Kumar, Peter Pushko, Gale Smith, Terrence M. Tumpey, Ted M. Ross

**Affiliations:** 1 Novavax, Inc., Rockville, Maryland, United States of America; 2 Center for Vaccine Research, University of Pittsburgh, Pittsburgh, Pennsylvania, United States of America; 3 Influenza Division, Centers for Disease Control and Prevention, Atlanta, Georgia, United States of America; Federal University of São Paulo, Brazil

## Abstract

**Background:**

Vaccination is a cost-effective counter-measure to the threat of seasonal or pandemic outbreaks of influenza. To address the need for improved influenza vaccines and alternatives to egg-based manufacturing, we have engineered an influenza virus-like particle (VLP) as a new generation of non-egg or non-mammalian cell culture-based candidate vaccine.

**Methodology/Principal Findings:**

We generated from a baculovirus expression system using insect cells, a non-infectious recombinant VLP vaccine from both influenza A H5N1 clade 1 and clade 2 isolates with pandemic potential. VLPs were administered to mice in either a one-dose or two-dose regimen and the immune responses were compared to those induced by recombinant hemagglutinin (rHA). Both humoral and cellular responses were analyzed. Mice vaccinated with VLPs were protected against challenge with lethal reassortant viruses expressing the H5N1 HA and NA, regardless if the H5N1 clade was homologous or heterologous to the vaccine. However, rHA-vaccinated mice showed considerable weight loss and death following challenge with the heterovariant clade virus. Protection against death induced by VLPs was independent of the pre-challenge HAI titer or cell-mediated responses to HA or M1 since vaccinated mice, with low to undetectable cross-clade HAI antibodies or cellular responses to influenza antigens, were still protected from a lethal viral challenge. However, an apparent association rate of antibody binding to HA correlated with protection and was enhanced using VLPs, particularly when delivered intranasally, compared to rHA vaccines.

**Conclusion/Significance:**

This is the first report describing the use of an H5N1 VLP vaccine created from a clade 2 isolate. The results show that a non-replicating virus-like particle is effective at eliciting a broadened, cross-clade protective immune response to proteins from emerging H5N1 influenza isolates giving rise to a potential pandemic influenza vaccine candidate for humans that can be stockpiled for use in the event of an outbreak of H5N1 influenza.

## Introduction

Vaccination is a potent and cost-effective counter-measure to the threat of seasonal or pandemic outbreaks of influenza [1]. The influenza virus is among the most devastating viral diseases due to the ease of spread as an aerosol and ability to cause severe sickness and mortality to susceptible humans. Currently licensed seasonal influenza vaccines are only partially protective, particularly in populations at highest risk of severe disease, the very young and the elderly. In addition, there is a need for novel approaches for enhancing immune responses to emerging influenza isolates of avian origin harboring a potential of causing an influenza pandemic outbreak that could infect and kill a considerable number of humans over a short period of time. Enhanced immunity is particularly important for vaccines protecting against such emerging strains, since pre-clinical and clinical studies have shown that some of these antigens such as those from H5N1 viruses are less immunogenic than antigens from seasonal influenza subtypes (Reviewed in: [1]–[4]). Recent research, however, has shown improved immunogenicity of some H5N1 antigens if supplemented with proprietary adjuvants [5]. If such approaches are shown to be well-tolerated in humans, they might also be able to stretch the limited supply of currently stockpiled vaccines. However, more research is needed in the discovery of novel vaccines, adjuvants, and dosing regimens to be able to supply the world with a safe and effective vaccine against avian influenza viruses.

The next influenza pandemic may be caused by an avian H5N1 influenza subtype virus [1]. In 1997, 18 confirmed cases of human infection with avian influenza A H5N1 viruses were identified in Hong Kong that resulted in six deaths [6]. These cases represented the first confirmed human outbreak associated with H5N1 influenza virus infection and raised global concerns about the occurrence of an influenza pandemic. This event led to intensive epidemiological monitoring of potential avian virus infection(s) by the World Health Organization (WHO) influenza surveillance network. Since 2003, outbreaks of avian influenza A (H5N1) have emerged and spread throughout southeast and central Asia, the Middle East, Africa, and Europe. Human cases increased between 2004 through 2007, with some H5N1 isolates showing resistance to antiviral drugs amantadine and rimantadine [7]–[10]. In addition, a second clade of H5N1 (represented by A/Indonesia/05/2005) has been identified with several subclades. Clade 2 isolates are genetically and antigenically distinct from clade 1 isolates (*i.e.* A/Viet Nam/1203/2004). In addition, there is evidence of limited human-to-human transmission by new isolates H5N1 influenza [11].

In August, 2006, the WHO advised that the choice of H5N1 strains for development of candidate vaccines should be representative of the distinct groups (clades) of viruses that have been afflicting humans recently [12]. The recent 2005–2006 outbreaks in Indonesia that were from clade 2 H5N1 viruses have already resulted in more than 50 human deaths and infected poultry in 28 of Indonesia's 33 provinces [13]. This raises the need for new studies to assess safety, immunogenicity, priming, cross-reactivity and cross-protection of vaccines against a H5N1 clade 2 virus.

To meet the demand for pandemic influenza preparedness and surge capacity following a newly identified pandemic influenza outbreak, our research group has developed a non-infectious influenza virus-like particle (VLP) platform for emerging isolates with pandemic potential [14]. These influenza VLP vaccines do not require the use of any live influenza virus during the development, manufacturing, or administration of these vaccines. As an alternative to conventional egg-based and mammalian cell-produced influenza vaccine approaches, these recombinant-based VLP vaccines are produced with a baculovirus system, which is a promising, innovative technology for efficient, safe, high-yielding and low-cost commercial vaccines for influenza virus.

Recently, our group described the development of influenza A H3N2 and H9N2 VLP vaccines comprised of only three influenza virus proteins, hemagglutinin (HA), neuraminidase (NA), and matrix 1 (M1) [14], [15] expressed in insect cells. These vaccines elicited high-titer antibodies that were efficacious in mice and ferret models [14], [15]. In this study, using a similar approach, VLP vaccines were constructed for from H5N1 isolates represented by A/Viet Nam/1203/2004 (clade 1) and A/Indonesia/05/2005 (clade 2). These investigational pandemic influenza vaccines were composed of non-infectious, non-replicating VLPs that maintained viral hemagglutination and neuraminidase activities. Each vaccine candidate was evaluated in mice for the ability to elicit an humoral and cell-mediated immune response in dose-sparing experiments, without the addition of adjuvants, and compared to those induced by baculovirus-derived recombinant HA (rHA) proteins. Vaccination with H5N1 VLPs showed protection against homologous challenge, as well as cross-clade protection from viruses representing both H5N1 clades 1 and 2 avian influenza.

## Materials and Methods

### Propagation of H5N1 reassortant viruses

The H5 HA and N1 NA of the reassortant H5N1 2005 viruses were derived from influenza A/VN/1203/2004 (VNH5N1-PR8/CDC-RG; termed VN/04) and A/Indonesia/05/2005 (Indo/05/2005(H5N1)/PR8-IBCDC-RG2; termed Indo/05) viruses and the internal protein genes came from the A/Puerto Rico/8/1934 (PR8) donor virus (kindly provided by Ruben Donis, Influenza Division, Centers for Disease Control and Prevention, Atlanta, GA, USA). Each virus requires the addition of 0.5 µg/ml TPCK-treated typsin to induce plaques in minimal essential medium (MEM) containing 0.8% agarose on chick embryo fibroblasts (CEF) or MDCK cells, as determined by Ruben Donis at the CDC. These reassortant viruses administered intranasally are not pathogenic to chickens (Ruben Donis, CDC, personal communication) or ferrets (personal observation). However, we determined that when these viruses (Indo/05; 1.8×10+5 pfu/ml and VN/04; 1.6×10+4 pfu/ml) were administered in 50 ul volume to anesthetized 8 week old BALB/c mice, both viruses caused severe weight loss within 8 days and 100% of mice died from complications associated with viral infection. Therefore, we used these viruses as challenge viruses for assessment of vaccine efficacy.

Virus stocks for the reassortant viruses were propagated in the allantoic cavity of 9- to 11-day-old embryonated specific pathogen-free (SPF) hen's eggs at 37°C. The allantoic fluids from eggs inoculated with each virus was harvested 24 h post-inoculation and tested for hemagglutinating activity. Eggs inoculated with reassortant viruses were incubated at 33°C and were harvested 3 days post-inoculation. Infectious allantoic fluids were pooled, divided into aliquots, and stored at −80°C until used for studies. The 50% tissue culture infectious dose (TCID_50_) for each virus was determined by serial titration of virus in Madin-Darby canine kidney (MDCK) cells and calculated by the method developed by Reed and Muench [16]. All experiments, including animal studies with infectious reassortant viruses, were conducted using enhanced BSL-2 containment procedures in laboratories approved for use by the USDA and Centers for Disease Control and Prevention. Animal experiments were approved by the National Institutes of Health Animal Care and Use Committee.

### Plaque Assay with and without Trypsin

MDCK cells plated in 6-well tissue culture plates were inoculated with 0.1 ml of virus serially diluted in Dubecco's modified Eagle's medium (DMEM). Virus was adsorbed to cells for 1 h, with shaking every 15 min. Wells were overlaid with 1.6% w/v Bacto agar (DIFCO, BD Diagnostic Systems, Palo Alto, CA, USA) mixed 1∶1 with L-15 media (Cambrex, East Rutherford, NJ, USA) containing antibiotics and fungizone, with or without 0.6 µg/ml trypsin (Sigma, St. Louis, MO, USA). Plates were inverted and incubated for 2–3 days. Wells were then overlaid with 1.8% w/v Bacto agar mixed 1∶1 with 2× Medium 199 containing 0.05 mg/ml neutral red, and plates were incubated for two additional days to visualize plaques. Plaques were counted and compared to uninfected cells.

### Cloning of HA, NA, and M1 genes and the generation of recombinant baculoviruses

**Figure 1:**
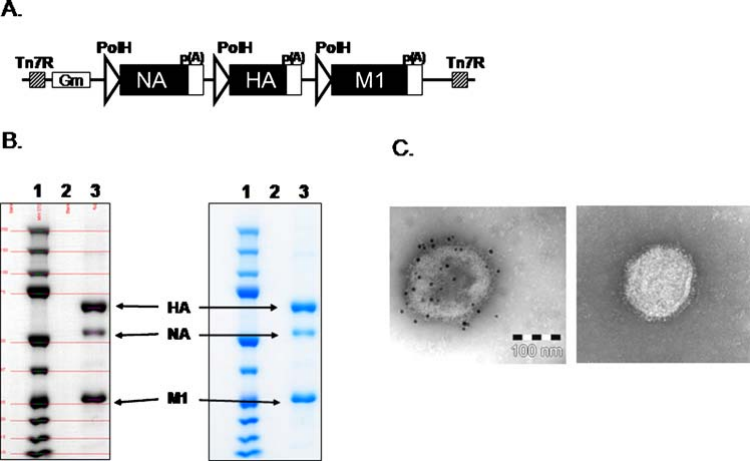
Expression of H5N1 virus-like particles. A) Baculovirus construct for expression of influenza A/Indonesia/05/2005 (H5N1) VLPs. Indicated are the polyhedrin promoters (PolH), polyadenylation signals, Tn7 regions, gentamicin resistance gene (Gm), and influenza genes (HA, hemagglutinin, M1, matrix 1 protein, NA, neuraminidase); B). Scanning densitometry analysis of purified Indo/05 VLPs. A sample of purified VLPs (4 µg) was electrophoresed on 4–12% polyacrylamide gel and stained with Coomassie blue (right panel, lane 3). A scanned image (left panel, lane 3) was used to determine the relative optical density (OD) of HA, NA, and M1. Purity is = OD HA+NA+M1/OD Total in the lane. Purity of this lot of Indo/05 VLPs was 96%. The location of HA, NA, and M1 structural proteins are marked. C). Immunogold electron microsopy of purified Indo/05 VLPs. Left Panel. Primary antibody: Influenza A H5N1 Anti-HA antibody (Biodesign). Secondary antibody: Goat anti-rabbit conjugated to 10 nm gold beads. Right Panel. Control antibody and goat anti-rabbit secondary antibody conjugated to 10 nm gold beads. Bar represents 100 nm scale.

The HA, NA, and M1 genes for the H5N1 VLP vaccine were synthesized by GeneArt (Germany) based upon sequences ISDN125873, ISDN125875, ISDN125876 [17] and followed by cloning into *E. coli* bacmids (Fig. 1A), plaque-purification of recombinant baculoviruses with HA, NA, and M1 genes in a single vector and expression in *Spodoptera frugiperda* Sf9 insect cells (ATCC CRL-1711) as previously described [14]. At 72 h post-transfection, cells were harvested for VLP production and recovery of recombinant baculoviruses in the culture medium. Particle expression was analyzed by sucrose gradient ultracentrifugation and SDS-PAGE followed by Western blot and purification of VLPs were essentially as previously described [14]. Indo/05 rHA (Lot # 31-10-06) was purified from the supernatants of Sf9 insect cells in-house and VN/04 rHA (Lot # 15-06-06) was purified from the supernatants of Sf9 insect cells acquired from Protein Sciences Corp., Meriden, CT, USA. Vaccines and protein were stored at 4°C prior to use.

### Baculovirus infections and purification of rHA and VLPs

Spodoptera frugiperda (Sf9) insect cells (ATCC CRL-1711) were maintained as suspension cultures in HyQ-SFX insect serum free medium (HyClone, Logan, UT, USA) at 28°C. Plaque isolates expressing influenza proteins were amplified by infecting Sf9 cells seeded in shaker flasks at 2×10+6 cells/ml at a multiplicity of infection (MOI) = 0.05. At 72 h post-infection, culture supernatants containing the recombinant baculoviruses were harvested, clarified by centrifugation, and stored at 4°C. Titers of recombinant baculovirus stocks were determined by agarose plaque assay in Sf9 cells.

Particle expression was analyzed by sucrose gradient ultracentrifugation and chromatography followed by Western blot as described by Pushko *et al.* [14]. Briefly, Sf9 cells were infected in 200 ml volume for 72 h at a cell density of 2×10+6 cells/ml with recombinant baculoviruses at a MOI = 3. Expression was determined by SDS–PAGE using 4–12% gradient polyacrylamide gels (Invitrogen, Carlsbad, CA, USA) and Coomassie staining and by Western blotting using antigen-specific sera (Fig. 1B). H5N1 specific sera included rabbit polyclonal sera raised against influenza A/Indonesia/05/2005 virus. Indo/05 rHA was purified from Sf9 insect cells following baculovirus infection. Briefly, rHA was extracted with a non-ionic detergent from Sf9 cells then purified to >99% using ion exchange and affinity chromatography.

VLPs were purified from Sf9 cells infected with baculovirus vectors expressing Indo/05 HA, NA, and M1 or VN/04 HA, NA, and M1 at an MOI 3 pfu/cells. Seventy-two hours post-infection, cultures were clarified to remove the cells and supernatants containing VLPs were concentrated by tangent flow filtration with a 500,000 molecular weight hollow fiber filter, then the concentrate was diafiltered against PBS. The diafiltered concentrate was centrifuged for 18 hr at 5,000×g on 20–60% sucrose gradients and the particles containing primarily VLPs and baculoviruses were recovered from a band in the gradients at about 30% sucrose. Separation of VLPs from baculoviruses was performed using ion exchange chromatography. The purity of VLPs was measured by scanning densitometry of SDS-PAGE using OneDscan system (BD Biosciences, Rockville, MD, USA) (Fig. 1B). VLPs were also analyzed for particle formation and the presence of hemagglutinin. Samples were coated on carbon-coated gold grids, stained with 5% solution of Ammonium Molybdate and air dried. A Zeiss 902 CEM electron microscope, operated at 80 kV, and digitally acquired with an Olympus Soft Imaging Solutions, GMBH. Mega View II digital camera at 1280×1024 pixel resolution was used. HA spikes on the surface of 100–200 nm particles were confirmed using immunogold staining with rabbit anti-HA or control antibody as shown in Fig. 1C left and right panels, respectively.

### Single-radial-immunodiffusion (SRID) assay

A quantitative single-radial-immunodiffusion (SRID) assay was performed essentially as described by Wood, et al. [18]. Briely, VLPs (lot# 07-14-06-WV-1/2) was analyzed using standardized CBER reagents: CBER Antibody (rg A/Vietnam/1203/1204 Lot # S-APSI Feb 5 2005) and CBER Reference Antigen (RG A/Vietnam/1203/1204). The Indo rHA (lot #301P) and the Indo VLPs were treated with 1% Zwittergent detergent (Calbiochem, EMD Biosciences, San Diego, CA, USA) just prior use. VLP samples were diluted and allowed to diffuse overnight in 1% agarose containing a dilution the anti-HA sheep reference serum. The agarose gel was stained with Coomassie and the diameter (mm) of antigen-antibody precipitation rings were measured with a micro comparator.

### Animals and vaccinations

BALB/c mice (*Mus musculus*, females, 6–8 weeks of age) were purchased from Harlan Sprague Dawley, (Indianapolis, IN, USA). Mice, housed in microisolator units and allowed free access to food and water, were cared for under USDA guidelines for laboratory animals. For vaccination, mice were anesthetized with 0.03–0.04 ml of a mixture of 5 ml ketamine HCl (100 mg/ml) and 1 ml xylazine (20 mg/ml). Mice (8 mice per group) were vaccinated with 3 µg or 600 ng doses (based on HA content) of either rHA or purified VLPs. Vaccines and protein were stored at 4°C prior to use. Animals were monitored for survival and morbidity (i.e. weight loss, ruffling fur, inactivity) weekly during the vaccination regimen and each day during viral challenge.

Blood was collected from anethesized mice via the retro-orbital sinus. Blood was transferred to a tube containing a serum separator and clot activator and allowed to clot at room temperature. Tubes were centrifuged and sera was removed and frozen at −80±5°C. All procedures were in accordance with the NRC Guide for the Care and Use of Laboratory Animals, the Animal Welfare Act, and the CDC/NIH Biosafety in Microbiological and Biomedical Laboratories.

### ELISPOT assays

**Figure 2:**
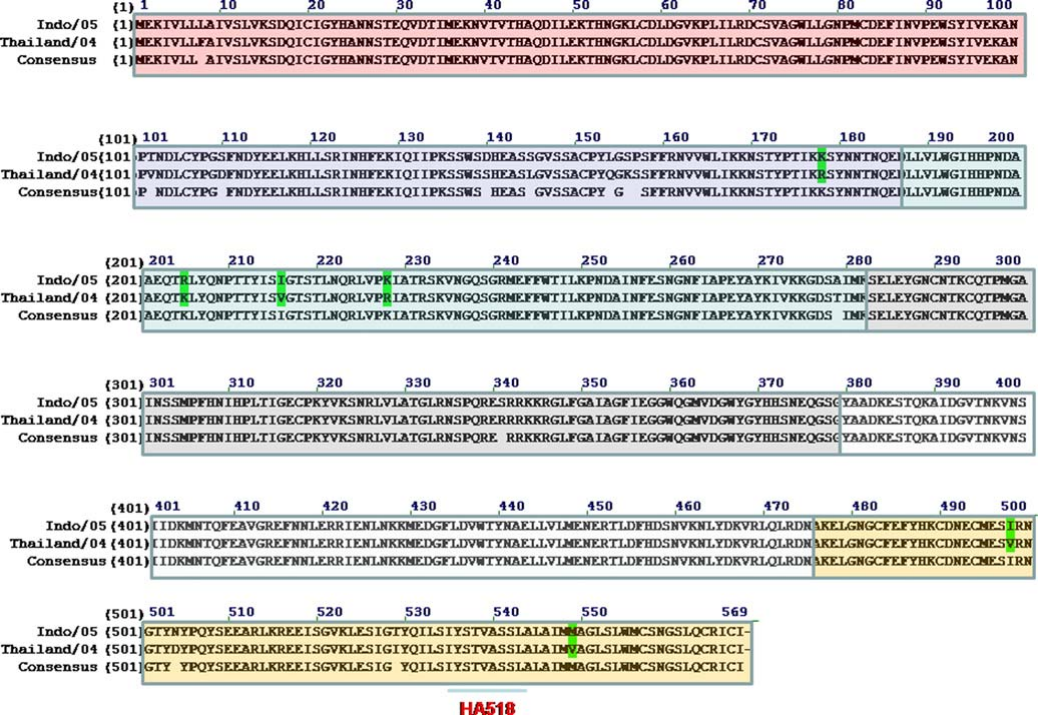
Amino acid sequence of hemagglutinin. Top line represents the sequence for A/Indonesia/05/2005, which is the isolate used to generate vaccines studied. Middle line represents the sequence for A/Thailand/2004, which is the isolate used to generate the peptides used for analysis. Bottom line represents the consensus amino acid sequence between the two sequences. Boxed, colored areas represent regions included in each of the 6 peptides pools (15-16 peptides per pool, 15mers overlapping by 11) used for stimulating splenocytes and lung cells in ELISPOTs. The location of the single H2-k^d^ peptide (HA518; (IYSTVASSL) is underlined in peptide pool 6.

Spleens and lungs were harvested (weeks 5 and 8) from vaccinated mice and cells were isolated for ELISPOT assays, as previously described [19], [20]. Briefly, cells were depleted of erythrocytes by treatment with ammonium chloride (0.1 M, pH 7.4). Following thorough washing with PBS, cells were resuspended in RPMI medium with 10% fetal bovine serum (cRPMI). Cell viability was determined by trypan blue exclusion staining. The number of anti-HA or anti-M1 specific murine IFN-γ (mIFN-γ) secreting cells was determined by enzyme-linked immunospot (ELISPOT) assay (R & D Systems, Minneapolis, MN, USA). Briefly, pre-coated anti-mIFN-γ plates were incubated (25°C for 2 h) with cRPMI (200 µl) and then were incubated with cell suspensions from the spleen or lungs (1×10^6^/well) isolated from vaccinated mice. Spleen or lung cell suspensions were stimulated (48 h) with pools of peptides (BEI Resources; 5ng/µl per peptide) representing the regions of influenza HA (Fig. 2) or M1 proteins. Additional wells of cells were stimulated with PMA (50 ng)/ionomycin (500 ng) or were mock stimulated. In addition, IL-2 was added to all wells (10 units/ml). Plates were washed with PBS-Tween (3X) and were incubated (25°C for 2 h) with biotinylated anti-mIFN-γ and incubated (4°C for 16 h). The plates were washed and incubated (25°C for 2 h) with strepavidin conjugated to alkaline phosphatase. Following extensive washing, cytokine/antibody complexes were incubated (25°C for 1 h) with stable BCIP/NBT chromagen. The plates were rinsed with dH_2_O and air dried (25°C for 2 h). Spots were counted by an ImmunoSpot ELISPOT reader (Cellular Technology Ltd., Cleveland, OH, USA).

### Serological assays

A quantitative ELISA was performed to assess anti-HA specific IgG or IgG isotypes in immune serum. Purified rHA (30 ng) was used to coat each well of a 96-well plate as previously described [21]–[24]. Plates were blocked (25°C for 2 hr) with PBS containing Tween 20 (0.05%) and nonfat dry milk (5%) and then incubated with serial dilutions of each serum sample (25°C for 2 hr). Following thorough washing in PBS-Tween 20 (0.05%), samples were incubated (25°C for 1 hr) with horseradish peroxidase (HRP) rabbit anti-mouse IgG (1∶5000) diluted in PBS-Tween 20 (0.05%) and nonfat dry milk (5%). The unbound antibody was removed, and the wells were washed. Strepavidin-HRP (1∶7000) was diluted in PBS-Tween 20 (0.05%) and incubated (25°C for 1 hr). Samples were incubated with TMB substrate (1 hr), and the colorimetric change was measured as the optical density (O.D., 405 nm) by a spectrophotometer (Dynex Technologies, Chantilly, VA, USA). The O.D. value of the age-matched naïve sera was subtracted from the samples using antisera from vaccinated mice. Results were recorded as the geometric mean titer (GMT)±the standard error (SE).

### Surface plasmon resonance (SPR) analysis

To assess the binding properties of serum antibodies, SPR technology was performed using a Biacore 3000 (Biacore AB, Uppsala, Sweden). Protein A (Pierce, Rockford, IL, USA) was immobilized to the surface of a CM5 sensor chip (Biacore, Inc., Piscataway, NJ, USA) using standard amine coupling chemistry. The surface of the chip was activated using a 1∶1 mixture of *N*-hydroxysuccinimide and 1-ethyl-3-(3-dimethyl aminopropyl) carbodimide hydrochloride (EDC) (Biacore, Inc.) followed by a 20-minute injection of protein A (70 µg/ml) to capture ∼5000 RU of protein A on two adjacent flowcells on a CM5 sensor chip. Remaining active carboxyl groups were inactivated with an injection of 1 m ethanolamine, and remaining non-covalently associated protein A was washed from the surface using four 30 sed injections of 100 mm HCl (100 µl/min). The second flowcell of the protein A-coated CM5 sensor chip was then used to capture polyclonal IgG antibody from VLP or rHA vaccinated mice. Serum was diluted in HBS-EP buffer such that a 5 µl injection at a flow rate of 10 µl/min yielded ∼100-300 RU total IgG antibody captured by the protein A on the CM5 chip. After capture of IgG, varying concentrations (0.7–300 nm, series of threefold dilutions) of rHA representing the Indonesia/05/2005 or Viet Nam/1203/2004 strains were passed sequentially over both flowcells of the sensor chip. A blank (0 nm) injection was also included. Binding isotherms were then analyzed using BiaEvaluation 4.1 (Biacore AB). It is important to realize that kinetic rates returned using these binding models for polyclonal serum represent only apparent rates of binding due to the multiple specificities inherent to a polyclonal response and do not define the kinetics of the polyclonal antibody anti-HA antibody response.

### Hemagglutination inhibition activity

The hemagglutination inhibition (HAI) assay was used to assess functional antibodies to the HA able to inhibit agglutination of horse erythrocytes. The protocol was adapted from the CDC laboratory-based influenza surveillance manual [1]. To inactivate non-specific inhibitors, sera were treated with receptor destroying enzyme (RDE) prior to being tested [21]–[23], [25], [26]. Briefly, three parts RDE was added to one part sera and incubated overnight at 37°C. RDE was inactivated by incubation at 56°C for ∼30 min and six parts PBS were added for a final 1:10 dilution of the sera. RDE-treated sera was two-fold serially diluted in v-bottom microtiter plates. An equal volume of reassortant virus, adjusted to approximately 8 HAU/50 µl, was added to each well. The plates were covered and incubated at room temperature for 20 min followed by the addition of 1% horse erythrocytes (HRBC) (Lampire Biologicals, Pipersville, PA, USA) in PBS. Red blood cells were stored at 4°C and used within 72 hours of preparation. The plates were mixed by agitation, covered, and the RBCs were allowed to settle for 1 h at room temperature [27]. The HAI titer was determined by the reciprocal dilution of the last row which contained non-agglutinated RBC. Positive and negative serum controls were included for each plate. All mice were negative (HAI≤10) for pre-existing antibodies to currently circulating human influenza viruses prior to vaccination [28].

### Isolation of cells from spleen and lung tissue

Spleens and lungs were excised, carefully rinsed with sterile PBS, and a single cell suspension was generated using a cell strainer (BD Biosciences, Bedford, MA, USA). Collected cells were centrifuged (1000×g for 5 min at 4°C). The cells were gently resuspended in 5 ml of RBC lysis buffer and incubated for 5 min at RT. PBS (5 ml) was added to each sample to neutralize RBC lysis buffer and then the cells were centrifuged (1000×g for 5 min at 4°C). The supernatants were discarded, and the cells were then resuspended in 3 ml of PBS. 200 µl of the cell suspension were aliquoted into 1.5 ml microcentrifuge tubes and stored at −80°C.

### Protection from lethal viral challenge

Mice that received either the VLP or rHA antigen were challenged with a lethal dose (10LD_50_) of one of the two H5N1 reassortant viruses. To determine the lethal dose of each reassortant H5N1/PR8 virus [16], mice were administered various dilutions of virus (50 µl) via the nares under one of two conditions: light or deep anesthetic. For light anesthetic, mice were administered a 1/10 dose of ketamine/xyalzine (as described above) that resulted in mice that were anesthetized for ∼5 minutes with light breathing. Mice returned to a normal state in ∼15 min. For deep anesthetic, mice were administered full dose of ketamine/xyalzine that resulted in mice being unconscious for greater than 30 min and the mice returned to a normal state in ∼1 h. Only, mice challenged with reassortant viruses under deep anesthetic showed signs of severe weight loss and mortality (data not shown). Mice were monitored daily for clinical signs of influenza infection and body weight was recorded each day. Mice that lost greater than 25% of body weight were euthanized. The ability of each vaccine to protect against homologous or heterologous challenge was compared to separate groups of naïve, unvaccinated control mice that were challenged with each reassortant virus.

Lung virus titers were determined using a plaque assay [29], [30]. Briefly, lungs from mice infected with virus were collected and single cell suspensions via passage through a 70 µM mesh (BD Falcon, Bedford, MA, USA) in 4 ml of PBS. Cell suspensions were frozen (−80°C) for 1 h, and then thawed, centrifuged at 1000×g for 10 min, and then the supernatants were collected and stored at −80°C.

Madin-Darby Canine Kidney (MDCK) cells were plated (5×10^5^) in each well of a six-well plate. Virus was diluted (1∶100 to 1∶1000) and overlayed onto the cells in 100 ul of DMEM supplemented with penicillin-streptomycin and incubated for 1 hr. Virus-containing medium was removed and replaced with 2 ml of L-15 medium plus 0.8% agarose (Cambrex, East Rutherford, NJ, USA) and incubated for 48 hrs at 37°C with 5% CO_2_. Agarose was removed and discarded. Cells were fixed with 70% EtOH, and then stained with 1% crystal violet for 15 min. Following thorough washing in dH_2_O to remove excess crystal violet, plates were allowed to dry, plaques counted, and the plaque forming units (pfu)/ml were calculated.

### Statistical analysis

Statistical analyses were performed using a two-tailed *t*-test with equal variance. Samples from VLP-vaccinated animals were compared to rHA-vaccinated animals and significance was considered at a *p*-value<0.05.

## Results

### Cell-mediated immunity elicited by VLP and rHA vaccines

**Figure 3:**
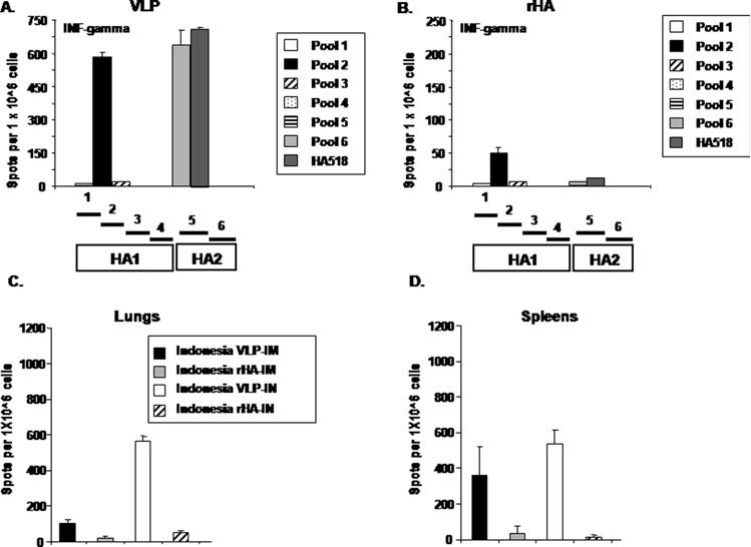
Elicitation of HA interferon-γ producing splenocytes and lung cells. ELISpots were performed on isolated splenocytes or lung cells from vaccinated mice (n = 8) collected at week 8. Cells (1×10^6^) were stimulated independently with pools of peptides representing different regions of HA. Splenocytes or lung cells were also stimulated independently with pools of peptides (15mers overlapping by 11 amino acids) or a single peptide HA518 (IYSTVASSL). Following stimulation, cells were assayed for mIFN-γ. HIV-1 Env peptides were used as a non-specific negative control. Splenocytes or lung cells stimulated with PMA/ionomycin were used as a positive control. (A) VLP vaccinated intramuscularly against all peptide pools, (B) rHA vaccinated intramuscularly against all peptide pools, (C). Lung responses using HA peptide pool 2. (D). Spleen responses using HA peptide pool 2.

**Figure 4:**
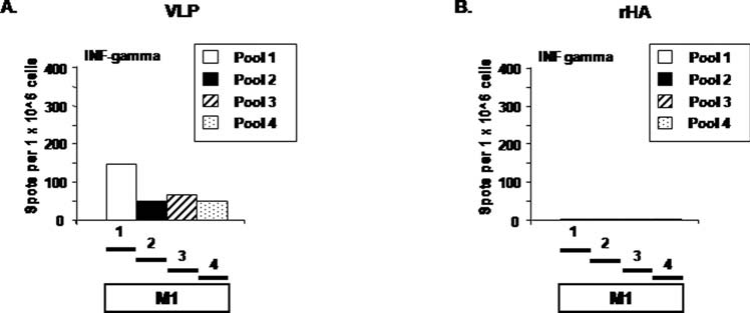
Elicitation of M1 interferon-γ producing splenocytes. ELISPOTs were performed as described in the legend to Fig. 3. Four pools (15mers, overlapping by 11) representing four regions of M1 were used to stimulate cells. HIV-1 Env peptides were used as a non-specific negative control. Splenocytes stimulated with PMA/ionomycin were used as a positive control. Number of IFN-γ ELISPOTs detected using each of the four M1 peptide pools from intramuscularly vaccinated mice with (A) VLP or (B) rHA vaccines.

Mice (BALB/c; n = 8) were vaccinated (week 0 and 3) via intramuscular injection or intranasal inoculation with purified influenza VLPs or purified rHA proteins representing the H5N1 isolate A/Viet Nam/1203/2004 (clade 1) or the A/Indonesia/05/2005 (clade 2) isolate. Collected splenocytes and lung cells were stimulated *in vitro* with pools of peptides specific for influenza H5N1 isolates (Fig. 2). Mice vaccinated with the influenza VLPs had a robust cell-mediated immune response against peptide pools from either the HA (Fig. 3) or M1 (Fig. 4). Cell-mediated immune responses were directed against epitopes in both the HA1 and HA2 subunits in mice vaccinated with VLPs, however, only peptides in pool 2 were recognized from rHA-vaccinated mice (Fig. 3A and B). Splenocytes stimulated with an immunodominant H-2^d^ peptide (HA518) contained in pool 6 had as strong a response as cells stimulated with the entire peptide pool 6. Only VLP-vaccinated mice had cellular responses to M1 (Fig. 4).

Mice vaccinated with VLPs intranasally had cell-mediated immune responses, as measured by mIFN-γ production, in both splenocytes and lung cells (Fig. 3C and D) however, mice vaccinated with VLPs intramuscularly had a robust cellular response in the spleen, but a limited cellular response in the lung. There was a limited or undetectable cellular response from isolated lung cells or splenocytes following vaccination with rHA protein (intranasally or intramuscularly). The number of mIFN-γ secreting splenocytes from age-matched mice, as well as splenocytes from mice vaccinated with VLPs and stimulated with an irrelevant peptide or unstimulated was negligible (10–12 spots) following *in vitro* re-stimulation (data not shown).

### Antibody responses to immunizations

**Figure 5:**
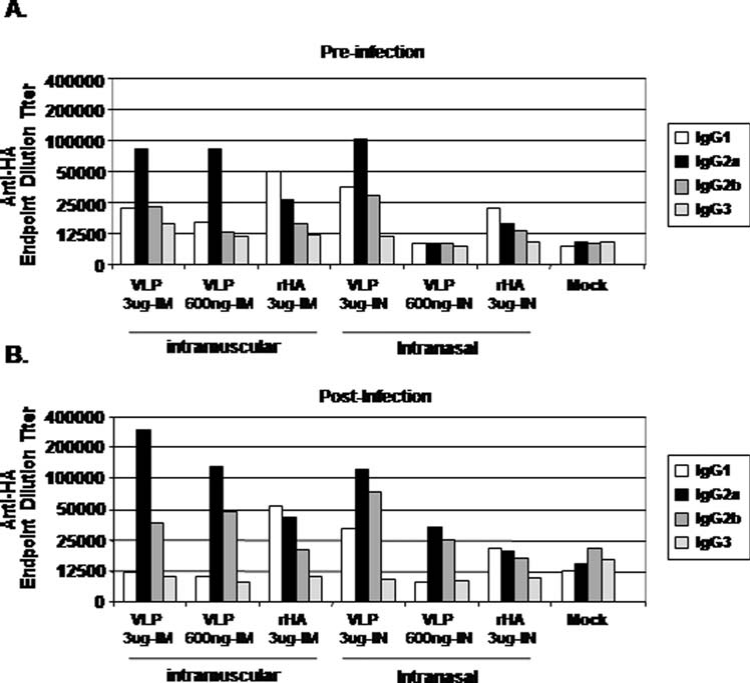
Anti-HA IgG Isotypes. The specific IgG isotype was assayed from the serum of each mouse group and the endpoint dilution titer was reported. (A) Week 5, pre-challenge. (B) Week 7, post-challenge. Each bar represents the average of 8 mice.

At week 5, mice vaccinated intramuscularly with VLPs or rHA had anti-HA antibodies that recognized the homologous rHA-coated plates with endpoint dilution titers that ranged between 10^4^-10^5^ (*i.e.* antibodies elicited by an Indonesia-derived vaccine binding to HA in an Indonesia rHA-specific ELISA) (Table 1). Mice vaccinated intranasally with Indonesia VLPs had an anti-HA endpoint dilution titer against Indonesia rHA that ranged between 10^5^-10^6^. The homologous anti-HA antibody titer in Viet Nam VLP vaccinated mice was 10^4^-10^5^, regardless of the route of inoculation. In contrast, anti-HA antibodies were low or undetectable in mice that received an intranasal inoculation of rHA antigen. Each serum sample was also tested for binding to the cross-clade HA in an ELISA. Interestingly, all serum samples that detected a homologous HA, also detected the heterologous H5N1 HA protein, albeit at a lower titer. As previously reported for H3N2 VLPs [15], the dominant serum IgG isotype subclasses elicited in VLP-vaccinated mice were IgG_2a _and IgG_2b_, indicative of a T helper type 1 response (Fig. 5A). In contrast to VLP vaccination, mice vaccinated with rHA elicited primarily an IgG_1_ (Th2) response. In a similar ELISA used to evaluate humoral responses directed to the NA, only mice vaccinated with VLPs had anti-NA antibodies (Table 2).

**Table 1:** Anti-HA Titer

		Intramuscular		Intranasal	
Clade	Vaccine (Dose)^a^	VN/1203/2004^b^	Indo/05/2005	VN/1203/2004	Indo/05/2005
**Clade 1-Vietnam**					
	**VLP (3 ug)**	**200,000** c	**12,500**	**200,000**	**12,500**
	**VLP (600 ng)**	**25,000**	**25,000**	**12,500**	**25,000**
	**rHA (3 ug)**	**6,400**	**12,500**	**200**	**<100**
	**rHA (600 ng)**	**800**	**3,200**	**<100**	**<100**
**Clade 2-Indonesia**	**VLP (3 ug)**	**12,500**	**200,000**	**400,000**	**2,000,000**
	**VLP (600 ng)**	**25,000**	**200,000**	**25,000**	**200,000**
	**rHA (3 ug)**	**3,200**	**12,500**	**<100**	**400**
	**rHA (600 ng)**	**1,600**	**6,400**	**<100**	**400**
	**Mock**	**<100**	**<100**	**<100**	**<100**

a Dose based upon HA content

b Isolate from which the HA was used.

c Anti-HA GMT titer.

**Table 2:** Anti-NA Titer.

		Intramuscular	Intranasal
Clade	Vaccine (Dose)^a^	Anti-NA	Anti-NA
**Clade 1-Vietnam**	**VLP (3 ug)**	**1600** b	**800**
	**rHA (3 ug)**	**<100**	**<100**
**Clade 2-Indonesia**	**VLP (3 ug)**	**3200**	**3200**
	**rHA (3 ug)**	**<100**	**<100**
	**Mock**	**<100**	**<100**

a Dose based upon HA content

b Anti-NA GMT titer at week 5.

### Vaccine induced hemagglutination-inhibition activity

Antibodies elicited by each vaccine were evaluated for the ability to inhibit virus-induced agglutination of horse red blood cells (Table 3). At week 5, 100% of the mice vaccinated with either clade 1 Viet Nam or clade 2 Indonesia VLPs had HAI titers ≥1∶40 against a reassortant virus containing HA and NA surface proteins matching the vaccine composition (homologous virus) (Table 3), regardless of the route of inoculation. Six of eight (75%) mice vaccinated intramuscularly with rHA had HAI titers greater than ≥1∶40, but none of the mice vaccinated intranasally with rHA had a measurable HAI titer. HAI titers elicited by Indonesia clade 2 VLPs against the clade 2 reassortant virus were >1 log higher than HAI titers elicited by Viet Nam clade 1 VLPs against the clade 1 reassortant virus. In contrast, serum from mice vaccinated with Indonesia clade 2 VLPs had >1 log lower HAI titers against the heterologous Viet Nam clade 1 reassortant virus compared to HAI titers against the homologous clade 2 reassortant virus, regardless the route of inoculation (Table 3). Viet Nam VLPs elicited similar HAI titers against both the homologous clade 1 reassortant virus, as well as the heterologous clade 2 reassortant virus.

**Table 3:** Hemagglutination-Inhibition Titer

		Intramuscular		Intranasal	
Clade	Vaccine (Dose)^a^	VN/1203/2004^b^	Indo/05/2005	VN/1203/2004	Indo/05/2005
**Clade 1-Vietnam**	**VLP (3 ug)**	**400±65** c *	80±53**	**256±54**	**121±20**
	**VLP (600 ng)**	**121±20**	**44±10**	**30±16**	**30±29**
	**rHA (3 ug)**	**46±15**	**10±0**	**10±0**	**10±0**
	**rHA (600 ng)**	**139±45**	**22±6**	**10±0**	**10±0**
**Clade 2-Indonesia**	**VLP (3 ug)**	101±27**	3377±1599*	80±53*	3377±809**
	**VLP (600 ng)**	**60±10**	845±202**	**12±2**	**1940±314ˆ**
	**rHA (3 ug)**	**10±0**	**211±100**	**10±0**	**10±0**
	**rHA (600 ng)**	**10±0**	**160±0**	**10±0**	**10±0**
	**Mock**	**10±0**	**10±0**	**10±0**	**10±0**

a Dose based upon HA content

b Influenza viruses used in the HAI assay against sera collected at week 5.

c HAI GMT plus or minus SEM.

d VLP vs. rHA comparing same dose, t-test, *p<0.05, **p<0.01, ˆp<0.001

### Protection against heterologous H5N1 viral challenge

**Figure 6:**
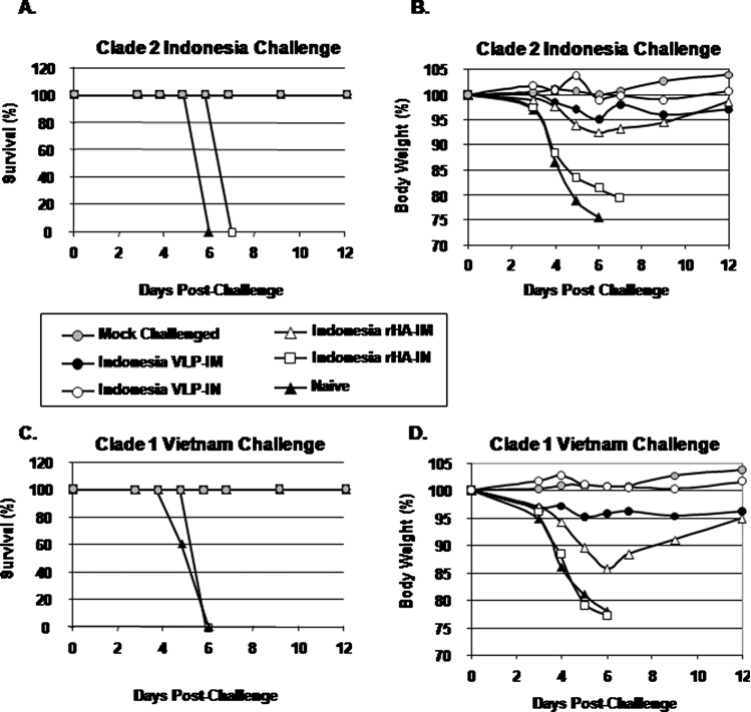
Protection from influenza virus challenge. At week 5, mice vaccinated with H5N1 clade 2-dervied vaccines were challenged intranasally with a lethal dose of reassortant influenza virus (A/Viet Nam/1203/2004 (clade 1) or A/Indonesia/05/2005 (clade 2)) and monitored daily for weight loss and mortality. The data are plotted as percentage of the average initial weight. Percentage of (A) original weight or (B) survival following challenge with clade 2 AIndonesia/05/2005 reassortant virus. Percentage of (C) original weight or (D) survival following challenge with clade 1 A/Viet Nam/1203/2004 reassortant virus. Mice that lost greater than 75% body weight were euthanized. Naïve mice were unvaccinated.

Mice that received either H5N1 clade 1 or clade 2 vaccines or unvaccinated control mice were challenged intranasally with a predetermined lethal dose of either a reassortant homologous or heterologous virus to evaluate the protective efficacy of each vaccine candidate. All mice vaccinated intramuscularly or intranasally with either VLP vaccine or intranasally only with rHA vaccines were protected from death following the lethal challenge of homologous virus whereas, all non-vaccinated, naïve mice lost greater than 25% of their original body weight and died from complications associated with infection by day 6 post-challenge (Fig. 6). Mice vaccinated with either VLP vaccine lost less than 5% of their original weight by day 6 following homologous viral challenge, regardless of the route of inoculation. In addtion, all VLP-vaccinated mice survived challenge, with little weight loss, following heterologous challenge (*i.e.* Indonesia clade 2 VLP vaccinated mice were protected against Viet Nam clade 1 challenge). However, there was more pronounced weight loss in mice vaccinated intramuscularly with either of the H5N1 rHA antigens following heterologous challenge, even though all mice survived. By day 21 post-challenge, all mice recovered to their original body weight, despite little or no detectable serum HAI antibodies against heterologous H5N1 virus prior to challenge (Table 3). In contrast, all mice vaccinated intranasally with rHA showed considerable weight loss and mortality similar tonaïve unvaccinated animals. Despite lower HAI titers compared to mice vaccinated with clade 2 vaccines, similar results were observed with mice vaccinated with Viet Nam VLPs and rHA vaccines (data not shown).

### Binding characteristics of serum antibody as determined by surface plasmon resonance

**Figure 7:**
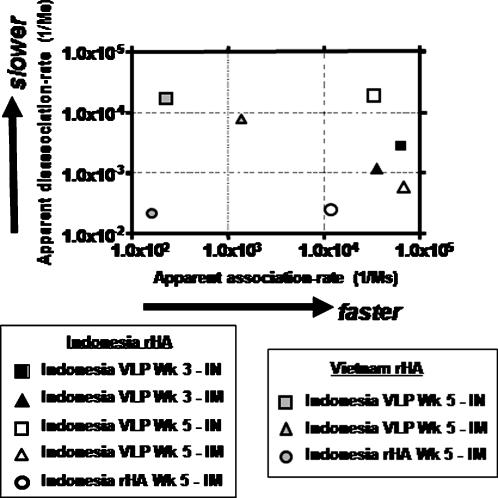
Kinetics of Indonesia VLP elicited antisera binding to H5N1 rHA antigens. Kinetics of antisera binding to homologous, Indonesia clade 2 rHA (black square) and heterologous, Viet Nam clade 1 rHA (white square). Values on x-axis represent the apparent association rate of antibody binding to HA and the values on the y-axis represent the apparent disassociation rate of antibody from the HA antigen.

In order to determine binding characteristics of polyclonal serum antibodies using surface plasmon resonanace (SPR), recombinant HA proteins, representing both H5N1 clades, were characterized (Fig. 7). Serum samples were diluted, polyclonal IgG was captured, and binding experiments were carried out as previously described [31], [32]. At week 5 post-vaccination, mice vaccinated intranasally with VLPs had a more dynamic pattern of antibody responses against the homologous HA antigen than mice vaccinated intramuscularly with the same vaccine (Fig. 7). Mice vaccinated with VLPs had one population of antibody that bound specifically to each homologous HA at an apparent association rate (*k*_a1_) that ranged from 1.55×10^4^ to 1.65×10^5^, regardless of the vaccine strain (Viet Nam clade 1 or Indonesia clade 2) or the route of vaccination (IM or IN). This apparent association rate was similar to antibodies elicited by rHA following intramuscular injection (1.13×10^4^). In contrast, the apparent dissociation rate (*k*_d1_) differed between intranasally or intramuscularly vaccinated mice against homologous HA. Following the boost, mice vaccinated intranasally with Indonesia clade 2 VLPs had antibodies with an apparent dissociation rate of 2.12×10^−5^ compared to sera from intramuscularly VLP-vaccinated mice that had a dissociation rate of 1.71×10^−3^, which was similar to the dissociation rate from mice vaccinated intramuscularly with Indonesia clade 2. rHA (4.11×10^−3^) (Fig. 7). Surprisingly, there was little change in the dissociation rate of VLP elicited antibodies when tested against the heterologous Viet Nam clade 1 HA antigen (3.01×10^−4^), but a significant decline in the association rate to the Viet Nam clade 1 HA (2.46×10^2^) compared to the Indonesia clade 2 HA. Similar results were observed with sera from mice vaccinated with Viet Nam clade 1 VLPs or rHA vaccines (data not shown). Therefore, the ability of antibody to bind to the heterologous HA antigen appears to be associated with the apparent association rate compared to antibody binding to the homologous HA antigen.

### Single vaccination elicits protection against challenge

**Figure 8:**
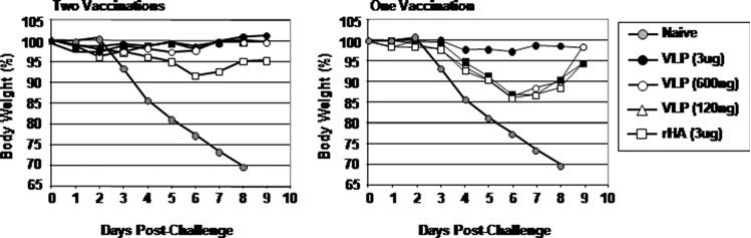
Protection from influenza virus challenge. At week 5, mice vaccinated with clade 2-dervied vaccines were challenged intranasally with a lethal dose of reassortant influenza virus (A/Indonesia/05/2005 (clade 2)) and monitored daily for weight loss and mortality. The data are plotted as percentage of the average initial weight. Percentage of original weight (A) Mice vaccinated with two doses of vaccine (week 0 and 3) and (B) mice vaccinated with a single dose of vaccine (week 0). All mice challenged at week 5. Mice that lost greater than 25% body weight were euthanized.

To determine if a single vaccination could elicit protective immunity to a lethal viral challenge, a second set of mice were vaccinated with a single intramuscular immunization (week 0) with purified influenza Indo/05 VLPs or rHA proteins and the responses were compared to mice vaccinated with two immunizations (week 0 and 3). Mice vaccinated with a single immunization of VLPs or rHA had no detectable HAI titers during the 5 week regimen (data not shown), but survived a challenge with 10LD_50_ of homologous reassortant Indonesia clade 2virus. Interestingly, mice vaccinated with the 3 ug dose of VLP had little to no weight loss (Fig. 8), which was similar to mice vaccinated with two doses of VLP or rHA vaccines (Fig. 4A). However, mice vaccinated with lower doses of VLPs or rHA lost ∼15% of their body weight by day 6 post-challenge, but recovered by day 10 post-challenge (Fig. 8). Naïve mice lost 80% of their body weight by day 5 post-challenge and all mice died from challenge by day 8.

Additional groups of vaccinated mice were euthanized on day 3 post-challenge and virus titers were determined from homogenized lung tissue (Table 4). Mice vaccinated with two doses of VLPs (3 ug) had no detectable Indo/05 virus (dilution 1:100) and no gross signs of infection. Unvaccinated naïve mice had an average titer of 1.56×10^6^ pfu/ml. These mice had ruffled fur, dyspnea, and lethargy by day 6 post-challenge. Sixty-six percent of the mice vaccinated with rHA (3 ug) had a detectable viral titer that was ∼3 logs lower than virus titers from unvaccinated mice (Table 4), but few gross pathological signs. Interestingly, mice vaccinated with a single immunization of VLPs had no detectable viral titers, whereas mice vaccinated with rHA had an average titer of 4.5×10^4^.

**Table 4:** Pathological and Virological Analysis.

Vaccine^a^	pfu/ml^b^	Weight^c^	Activity^d^	Dyspnea^e^	Survival^f^
**VLP (2 doses)**	**<1.00E+02**	**98%**	**0**	**0**	**100%**
**rHA (2 doses)**	**6.00E+03**	**91%**	**0**	**0**	**100%**
**VLP (1 dose)**	**<1.00E+02**	**97%**	**0**	**0**	**100%**
**rHA (1 dose)**	**4.50E+04**	**86%**	**1**	**0**	**100%**
**Mock**	**1.57E+06**	**80%**	**2**	**1**	**0%**

a Vaccine (3 µg) administered at weeks 0 (1 dose) or week 0 and 3 (two doses).

b Particle forming untis (pfu) per milliliter (ml) in the lungs of mice at day 3 post-challenge. <1.00e+2 = viral titers less than 100 pfu/ml.

c Percentage of original weight at day 6 post-challenge.

d Activity score. 0 = Full activity. 1 = slow to respond to touch, but still mobile. 2 = little response to touch.

e Dyspnea. 0 = no shortness of breadth. 1 = heavy breathing and shortness of breadth.

f Percentage of mice that survived challenge at day 8 post-challenge.

## Discussion

Influenza virus-like particle vaccines described in this report are non-replicating particle-based vaccine candidates for influenza based upon a influenza A H5N1 clade 1 and 2 isolates. Our results show that BALB/c mice immunized with VLP vaccines were effectively protected from disease and death when challenged with viruses with antigenically distinct HA and NA proteins from H5N1 influenza viruses. These results highlight the potential of VLP vaccine as an effective immunogen and delivery system for influenza antigens, particularly to the respiratory tract. Our VLPs have the advantage of inducing strong humoral and cellular immune responses against multiple influenza viruses without the need of a supplemental adjuvant. The inclusion of the highly conserved M1 protein is also advantageous, since CD8+ T cells against conserved epitopes within M1 can contribute to protection against morbidity and mortality from influenza [33]–[38]. Pools of peptides representing M1 were used to identify cellular responses elicited by our VLPs (Fig. 4). In addition, these vaccines have the potential to elicit protective immune responses as effectively as live-attenuated influenza without safety issues associated with the isolation, production, and delivery of live vaccines [4].

Currently licensed seasonal influenza vaccines elicit immunity that is subtype- and sometimes strain-specific and do not protect against avian H5N1 viruses with pandemic potential. Our influenza VLPs are a new generation of egg-independent candidate vaccines expressed from insect cells infected by a recombinant baculovirus that encodes genes for three influenza virus proteins, HA, NA, and M1 [14], [15]. These VLPs may have an advantage over HA-only based vaccines by the inclusion of these additional viral proteins, especially against evolving H5N1 isolates from various clades. These VLPs elicited anti-NA antibodies against an N1 NA protein that was not matched to the vaccine. Antibodies against the same subtype of NA, but not the exact NA molecule, can contribute to protective immune responses [39]. When delivered via parenteral or mucosal routes, VLPs may be particularly effective immunogens at priming T cells and targeting antigen-presenting cells, as described for other VLPs [40]–[43], as well as inducing high titered antibody responses [44], [45]. Another key factor is the authentic presentation of surface HA and NA in native, three-dimensional conformation. Recent clinical trials of human papillomavirus (HPV) VLPs have led to FDA approval [46], [47] and therefore, this may bode well for the approval of additional VLP-based vaccines, including influenza VLPs. Our influenza VLPs are easy to develop, produce, and manufacture. They are not labor-intensive and they do not require costly production schemes typically associated with manufacturing vaccines in eggs. VLP vaccines, like other recombinant influenza vaccines, are particularly advantageous to address future pandemics because these vaccines 1) need shorter lead times for development of vaccines matched to circulating strains of viruses, 2) use recombinant DNA technology to alleviate safety restrictions and bottlenecks associated with dependence on live viruses, 3) use cell culture based methods with disposable bioreactors to provide rapid response and higher yields (scalable and transferable) for improved surge capacity.

In studies reported here, all mice vaccinated intramuscularly with either vaccine or intranasally with the VLP vaccines survived challenge with lethal doses of reassortant viruses. However, intranasal delivery of the VLPs did elicit a broader immune response than the same vaccine delivered intramuscularly. Cellular responses, in particular, were reduced in the lung mucosa in mice vaccinated intramuscularly compared to intranasally vaccinated mice. The ability to elicit mucosal immune responses in the respiratory tract, including the lungs, is desirable for an influenza vaccine. Neutralization of influenza by pre-existing sIgA and IgG in the lung reduces infection of susceptible epithelial cells [48]–[50] and thereby reduces the deleterious effects induced by elevated cytokine levels, which typically lead to the development of fever and respiratory symptoms [51]–[53]. H5N1 infection in humans activates cytokine/chemokine secretion resulting in the occurrence of a “cytokine storm” that may contribute to the severity of disease by these viruses [53]–[56]. The levels of these pro-inflammatory cytokines, triggered by influenza gene products, are higher during H5N1 virus infection compared to seasonal influenza virus infection [57]. Therefore, vaccines, such as VLPs studied here, that prevent infection by antibody or quickly clear infected cells by cell-mediated immune responses [58]–[63] in the lung mucosa may blunt the activation of this deleterious immune activation by reducing viral replication.

Compared to particulate antigens, intranasal vaccination of soluble proteins, in the absence of an adjuvant, induces low or undetectable immune responses in rodents and primates [41], [42], [64]. In the nasal mucosa, VLPs are most likely phagocytosed by microfold epithelial cells (M cells) in the nasal lumen and then directly deposited to the NALT (nasal associated lymphoid tissue) via M cell transcytosis [65], which preferentially drains into lymph nodes. This process induces strong local (NALT) and distant immune responses in both peripheral and mucosal immune compartments [66]. In contrast, soluble antigens can penetrate the nasal epithelium and directly interact with dendritic cells, macrophages and lymphocytes and then these antigens are transferred to posterior lymph nodes [67]. Soluble antigens can bypass the NALT and be directly fed into superficial lymph nodes by antigen presenting cells in the nasal lumen resulting in a lower local immune response [66]. Therefore, VLP immunogens can potentially interact directly with the mucosal immune system to elicit high titer immunity.

Several approaches are in progress to develop vaccines against H5N1 viruses. To date, products tested in humans have not been effective at producing a strong immune response in a large percentage of subjects tested in clinical trials [68]. However, many of these previous H5N1 vaccine candidates were derived from clade 1 or clade 3 isolates that required multiple doses and/or the use of various adjuvants to achieve levels of antibodies believed to correlate with seroprotection in a majority of subjects tested [34], [48], [69]–[78]. Results presented in this report indicate that our A/Indonesia/05/2005 (clade 2) VLP vaccine elicited higher HAI antibody titers than the A/Viet Nam/1203/2004 (clade 1) VLP vaccine without the use an adjuvant and elicited a robust and broadly reactive immune response following two vaccinations. Interestingly, a single immunization was able to protect mice from virus-induced death, albeit mice administered lower doses of VLPs or rHA had viral replication in the lungs and transient weight loss. These results are similar to recent live attenuated vaccines that required two vaccinations to prevent weight loss, but were able to protect ferrets following a single vaccination [4]. The ability to protect humans using a “one-shot” vaccination regimen is highly desirable for a vaccine against influenza isolates with pandemic potential. Following an outbreak, vaccines that reduce viral titers in the lung and nasal mucosa may slow the transmission of the virus among humans; there may not be sufficient time for a booster shot of vaccine to achieve optimal antibody titers. The VLP vaccine described in this report demonstrates that a one-dose regimen is potentially possible in rodents using a non-replicating immunogen that can elicit cross-clade protective immune responses and is worthy of evaluation in the clinic.

One of the challenges faced by influenza vaccine developers is the ability to protect populations in the face of a spreading pandemic. The next influenza pandemic may be caused by a H5N1 virus and if so, it is not known which clade or subclade will be responsible. Correlates for protection from infection by H5N1 isolates have not been determined. Historically, the HAI assay is the most widely used serological assay for monitoring influenza immunity and is the accepted standard for measuring functional influenza-specific serum antibodies to the hemagglutinin following vaccination. An HAI titer that is greater than 1:40 (≥40) against a seasonal influenza strain is believed to be protective for ∼50% of the vaccinated population [18]. However, this does not appear to hold true for avian H5N1 viruses, since no correlation between HAI titer and protective efficacy against H5N1 infection has been reported in animal or human systems. Therefore, new correlates may be necessary to assess the efficacy of potential H5N1 vaccines. One interesting finding in this study was the correlation between the slower disassociation rates of the VLP-elicited antibody to HA compared to antibodies produced in response to rHA vaccines. In addition, antibodies elicited to the homologous clade 2 rHA had faster association rates compared to antibody binding the heterologous clade 1 rHA. The increase in antibody association rates *in vivo* could bind HA on viruses quickly and perhaps decrease the number of infected cells in the lung, and thus could act to reduce the amount of viral replication to allow the immune system opportunity to better control the infection. In addition, antibodies that are slow to dissociate from the virion may continue to reduce the ability to uncoat and thus restrict the virus post-infection. Further analysis is needed; however, the use of antibody association/dissociation rates may be a more accurate assessment of vaccine efficacy that could potentially correlate with enhanced efficacy.

One of the more interesting findings in this study was the severity of disease induced by the 6:2 H5N1/PR8 reassortant viruses in the BALB/c mouse model. Previous publications using 1997 H5N1/PR8 clade 3 reassortant viruses generated by Subbarao and colleagues were not lethal for mice [79]. Therefore, we were expecting the clade 1 and clade 2 reassortant viruses to also be non-lethal and to be able to culture viruses from lungs of infected mice to compare the efficacy of each vaccine based only upon reduction of virus titers. Instead, we found both the clade 1 and clade 2 reassortant viruses to cause precipitous weight loss and to be lethal for mice. However, lethality was only observed in mice infected under deep anesthetic, since mice infected under lighter anesthesia conditions showed less dramatic weight loss (∼7%) and no mortality (data not shown). Therefore, we speculate that these reassortant viruses are lethal under conditions when the virus is allowed to infect the lower respiratory tract. Interestingly, we found that not all clade 2 H5N1/PR8 reassortant viruses were lethal to mice, since mice administered a similar high dose (10^6^ pfu/ml) of the PR8 reassortant viruses with HA and NA proteins from A/Anhui/1/2005 (clade 2.3) did not cause mortality. In addition, none of these reassortant viruses were lethal in a ferret model (data not shown), which may be a reflection of the difference in anatomy, sites of replication, and distribution of viral receptors between BALB/c mice and ferrets. We acknowledge that wild-type H5N1 isolates may result in more pathology in mice compared to the H5N1 HA/NA reassortant viruses used in this study. Many components of avian H5N1 isolates have been attributed to the highly pathogenic nature of these viruses, including the H5N1 PB2 and NS1 proteins, which are not included in the H5N1-PR8 reassortant viruses used here. Nonetheless, reassortant viruses can be useful tools to evaluate immune responses raised to H5N1 HA and NA components in the absence of a high containment facility and special permits required to work with wild-type H5N1 viruses. We will soon have additional capacity to be able to do future immunogencity studies against highly pathogenic wild-type H5N1 isolates.

This is the first report of an H5N1 VLP vaccine derived from a clade 2 influenza isolate. These clade 2 H5N1 VLP and rHA vaccines elicited protection against a lethal challenge from an antigenically similar reassortant virus strain without including an adjuvant. However, rHA only vaccines, administered at the same dose, did not prevent morbidity and weight loss following a cross-clade challenge even though all animals did eventually recover. In contrast, a 3 µg dose of VLPs provided cross-clade protection against a H5N1 challenge with little to no observed weight loss. It is reasonable to expect that use of VLP-based vaccines will provide substantial clinical protection and reduce mortality in humans and appropriate clinical studies should be initiated to further evaluate the potential of VLP vaccines for both seasonal and pandemic influenza.
